# Giant Wrinkles on the Surface of Epitaxial BaTiO_3_ Thin Films with Drastic Shrinkage during Transfer from a MgO(100) Single-Crystal Substrate to a Flexible Polyethylene Terephthalate Sheet

**DOI:** 10.3390/s21217326

**Published:** 2021-11-03

**Authors:** Hiroaki Nishikawa, Shinji Umatani, Tomofumi Mizuyama, Akihiro Hiraoka, Katsuhiro Mikami

**Affiliations:** 1 Faculty of Biology-Oriented Science and Technology, Kindai University, 930 Nishi-Mitani, Kinokawa 6496493, Wakayama, Japan; kmikami@waka.kindai.ac.jp; 2 Graduate School of Biology-Oriented Science and Technology, Kindai University, 930 Nishi-Mitani, Kinokawa 6496493, Wakayama, Japan; shinji.umatani@gmail.com (S.U.); 2033730016k@waka.kindai.ac.jp (T.M.); kindai.hiraoka@gmail.com (A.H.)

**Keywords:** giant wrinkle, shrinkage, transfer process, BaTiO_3_, flexible device

## Abstract

The transfer of ferroelectric and piezoelectric BaTiO_3_ epitaxial thin films from an original MgO(100) single-crystal substrate to a polyethylene terephthalate (PET) sheet has been studied to fabricate *flexible epitaxial functional oxides*. The outline of our previous transfer process is as follows: the epitaxial BaTiO_3_ thin films were deposited on the MgO(100). Then, the surface of the BaTiO_3_ was adhered onto a PET sheet. Finally, only the MgO(100) substrate was dissolved in a phosphoric aqueous solution, which resulted in the transfer of the epitaxial BaTiO_3_ thin film from the MgO(100) to a PET sheet. To establish this transfer process, our aim was to prevent any damage, such as cracks and exfoliation, during the transfer of the epitaxial functional oxides. We found that a Pt buffer layer with a ductile nature was effective for improving the quality of transferred epitaxial BaTiO_3_ thin films. Moreover, the epitaxial BaTiO_3_ thin films showed a drastic shrinkage of ca. 10%. The surfaces of the shrunk, epitaxial BaTiO_3_ thin films showed giant wrinkles with a micrometer-order amplitude and a 10-μm-order periodicity without any damage. The epitaxial BaTiO_3_ thin films with giant wrinkles, accompanied by drastic shrinkage, are similar to the thin films that are coated on a pre-stretched elastomer, which is one of the fabrication processes of stretchable devices.

## 1. Introduction

The development of thin film transistors, consisting of oxide semiconductors on flexible substrates, has extended the field of oxide electronics to flexible devices [1]. This first step of *flexible oxide engineering* uses an amorphous oxide grown at room temperature [2,3,4,5] because crystalline oxides, particularly multi-element systems, require higher process temperatures (generally more than 500 °C). Such high temperatures exceed the heatproof limit of most polymers, which are the typical materials used for flexible substrates. Amorphous oxides showing attractive functionalities are effective for flexible oxide engineering because most amorphous oxides grow even at room temperature. However, such appli- cation of functional oxides is not a traditional subject in the field of oxide electronics. Since the discovery of cuprate superconductors [6], one of the most studied subjects of oxide electronics has been the device applications of the characteristic properties, such as the superconductivity, ferroelectricity, piezoelectricity, ferromagnetism, and magneto-resis- tivity. Furthermore, for bilayer systems, advanced studies in this field have developed devices using the interaction between the functionalities of the two layers beyond the het- erointerfaces [7,8,9,10,11,12]. These studies have required the crystallization of oxides with appropriate structures, because these characteristic properties are accompanied by specific crystal structures. This means that the application of amorphous oxides to flexible oxide engineering is not a subject in the field of *traditional* oxide electronics, but it is an emerging new subject.

We aimed to create flexible oxide engineering of various traditional functional oxides. To achieve this goal, we have investigated the transfer process of epitaxial functional oxide thin films [13,14]. The outline of the transfer process is as follows: the functional oxide is grown on an original substrate that is suitable for epitaxy at a high process temperature, to prepare an epitaxial thin film with good crystallinity. In the next step, the thin film of the functional oxide is bonded to a polymer substrate. The epitaxial thin film with the polymer substrate is then released from the original substrate via one of several techniques [13,14,15,16,17,18,19,20,21,22,23,24]. During the process, the epitaxial thin film of the functional oxide is transferred from the original substrate to a polymer substrate. One of the most important goals of the transfer process is to prevent damage of the transferred thin film during its release from the original substrate [13,14]. This problem has limited the size of the transferred epitaxial functional oxide thin film in past studies [25,26].

In this study, we inserted a Pt buffer layer to relax the stresses on the origin of the damage for the transferred epitaxial BaTiO_3_ (a ferroelectric and piezoelectric material) thin film for a larger sized transfer. The enlargement of the transferred thin film without any damage is appropriate for the application of this process to practical manufacturing and conventional micro/nanopatterning techniques. In the process of enlarging the size of the transferred epitaxial BaTiO_3_ thin film with a Pt buffer layer, we found two remarkable phenomena of the transferred epitaxial BaTiO_3_ thin film: drastic shrinkage and giant wrinkles without any damage, despite the brittle nature of BaTiO_3_. Such giant wrinkles are similar to the surface structures observed after the relaxation of thin films that have been deposited on pre-stretched elastomer [27,28,29] to fabricate stretchable devices. Here, we report these interesting results of epitaxial BaTiO_3_ thin films.

## 2. Materials and Methods

**Figure 1:**
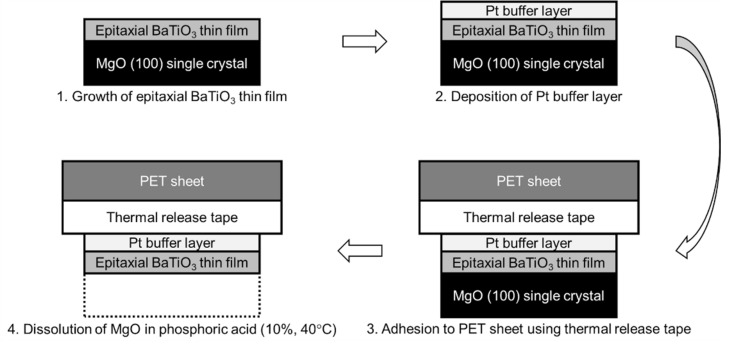
Schematic illustration of the process of transfer of an epitaxial BaTiO_3_ thin film to a flexible PET sheet. The additional step from our previous process [13,14] is the second step: deposition of a Pt buffer layer.

The transfer process of the epitaxial BaTiO_3_ thin films is schematically illustrated in Figure 1. BaTiO_3_ thin films were prepared on MgO(100) single-crystal substrates (10 mm × 10 mm × 0.5 mm, front-side polished, K&R Creation Co., Ltd., Ikeda, Japan) by pulsed laser deposition (PLD) with a KrF excimer laser (COMPex 102, Coherent Inc., Santa Clara, CA, USA, *λ* = 248 nm). During deposition, the MgO substrate was heated to 700 °C. Pure O_2_ gas with a partial pressure of 10^−2^ Pa was introduced into the growth chamber (custom ordered apparatus, base pressure 10^−5^ Pa, Seinan Industries Co., Ltd., Osaka, Japan). Because the growth chamber was evacuated from ordinary air to base pressure of 10^−5^ Pa, the residual gas is almost the same composition as that of air (N_2_ = ca. 80% ~ 8 × 10^−6^ Pa and O_2_ = ca. 20% ~ 2 × 10^−6^ Pa) and introduced O_2_ of 10^−2^ Pa was added. The parameters of the laser beam were a laser fluence of 1.5 J/cm^2^ and a spot area of 2.0 mm^2^ [30] on the sintered BaTiO_3_ target (99.9% purity, Kojundo Chemical Laboratory Co., Ltd., Sakado, Japan). The pulse repetition rate of the laser beam was 1 Hz (corresponding to a growth rate of 0.5 nm/min until the total thickness reached 60 nm), 3 Hz (corresponding to a growth rate of 1.5 nm/min until the total thickness reached 250 nm), and 5 Hz (corresponding to a growth rate of 2.5 nm/min until the thickness reached 600 nm) independent of the final thickness. The orientations and crystallinities of the BaTiO_3_ thin films were verified by conventional X-ray diffraction (XRD, Ultima 4, Rigaku Corporation, Akishima, Japan) using 2*θ*/*θ* measurement. The final thickness was measured with a stylus surface profiler (Dektak 150, Bruker Corporation, Santa Barbara, CA, USA). The deposited epitaxial BaTiO_3_ thin films were coated with a thin Pt layer (ca. 30 nm thick) by direct current sputtering (Quick Coater SC-701, Sanyu Electron Co., Ltd., Tokyo, Japan) after taking out the epitaxial BaTiO_3_ thin films from the growth chamber. The epitaxial BaTiO_3_ thin film on the MgO(100) single-crystal surface was not heated during sputtering, so the thin Pt layer was amorphous. The Pt/epitaxial BaTiO_3_ thin film on the MgO(100) single-crystal surface was bonded with thermal release tape (TR series, 40-μm-thick acrylic adhesive, Sumiron Co., Ltd., Osaka, Japan) to a polyethylene terephthalate (PET) sheet (100 μm thick). The whole sample was immersed in 10% phosphoric acid aqueous solution at 40 °C for several hours to dissolve the MgO(100) single crystal [14]. This process did not dissolve the other layers, and only the epitaxial BaTiO_3_ thin film bonded to the flexible PET sheet with the thermal release tape remained. The completion of the transfer process was verified by XRD measurement, i.e., disappearance of the MgO peaks. The surface morphology of the epitaxial BaTiO_3_ thin film was observed by confocal laser scanning microscopy (CLSM, LEXT OLS3100, Olympus Corporation, Tokyo, Japan).

## 3. Results and Discussion

### 3.1. Improvement of the Quality of Transferred Epitaxial BaTiO_3_ Thin Films: Effect of Insertion of a Thin Pt Buffer Layer between the Epitaxial Thin Film and PET Sheet

A problem with our previously reported process was serious damage (cracks and exfoliation) of the transferred epitaxial thin films [13,14]. We considered that the transferred epitaxial thin films were damaged by the tensile strain due to the difference between the thermal expansion of both BaTiO_3_ and PET, which were bonded with thermal release tape (made of polymethylmethacrylate). The coefficient of linear thermal expansion has been reported to be 6.00 × 10^−6^ to 7.52 × 10^−6^ K^−1^ for BaTiO_3_ [31,32,33], 56.67 × 10^−6^ to 72 × 10^−6^ K^−1^ for PET [34,35] and 66 × 10^−6^ to 90 × 10^−6^ K^−1^ for PMMA [36]. When the MgO substrate was etched, the temperature of the etchant (phosphoric acid aqueous solution) was kept at 80 °C [13] or 40 °C [14]. The heating of the etchant, to maintain the temperature, applied tensile strain to the BaTiO_3_ thin films, because of the much larger thermal expansion of PET and PMMA than BaTiO_3_. For example, in case of heating from 25 to 40 °C, the thermal expansions of PET and PMMA are ca. 0.1%, which is one order of magnitude larger than that of BaTiO_3_ (ca. 0.01%).

**Figure 2:**
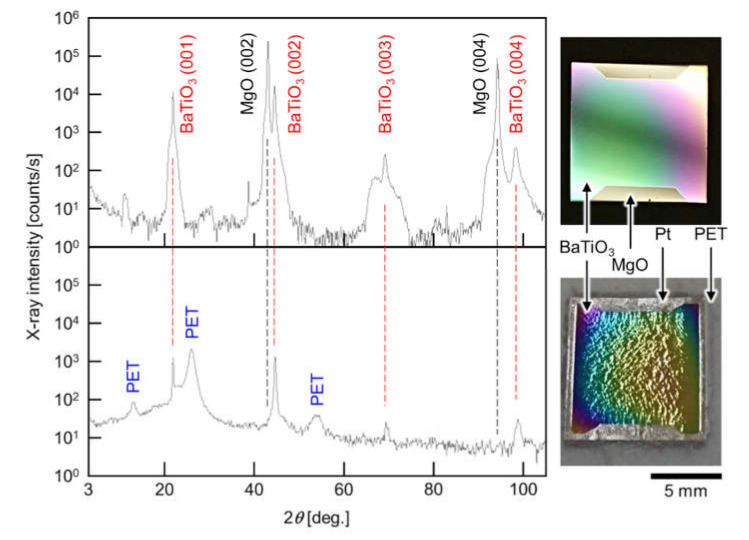
XRD patterns of the epitaxial BaTiO_3_ thin films before (**top**) and after (**bottom**) the transfer process. After transfer, the BaTiO_3_ peaks remained, but all of the MgO peaks disappeared. A photograph of the sample is shown on the right-hand side of each XRD pattern.

If the difference between the thermal expansion of PET and PMMA, and BaTiO_3_ is the origin of the damage of the transferred thin films, then a way to prevent damage is to absorb the tensile strain from the PET and PMMA to epitaxial BaTiO_3_ thin films. We proposed the insertion of a ductile Pt thin layer to absorb the tensile strain caused by the difference between the thermal expansion of PET and PMMA, and BaTiO_3_ (Figure 1). The modification of our previous method [13,14] is to sputter Pt at room temperature before the adhesion of the epitaxial BaTiO_3_ thin film to be transferred to the PET sheet with thermal release tape. The XRD patterns, and the photographs before and after the transfer of a 200-nm-thick epitaxial BaTiO_3_ thin film, are shown in Figure 2. From the XRD patterns, the transfer of the epitaxial BaTiO_3_ thin film was successful, namely, the (00*l*) peaks of BaTiO_3_ remained, but all of the peaks that were derived from the original MgO(100) substrate disappeared after the transfer. The lack of Pt peaks in the XRD patterns indicated that the Pt buffer layer was in an amorphous phase. The photographs clearly showed that the transferred epitaxial BaTiO_3_ thin film was not exfoliated even though it was relatively large (10 mm × 10 mm). As the sizes of the transferred oxide thin films without serious exfoliation in many previous studies were in the order of millimeters [13,14,15,17,20,24,25,26], the transfer of a 10 mm × 10 mm oxide thin films without serious exfoliation is a noteworthy result. This process is expected to be applied to the practical manufacturing of flexible oxide devices combined with nano/micropatterning techniques. Another feature is that the inserted Pt buffer layer also functions as a bottom electrode for the epitaxial BaTiO_3_ thin film. Thus, the improved transfer process in this study can be directly used for device applications.

### 3.2. Giant Wrinkles Accompanied by Drastic Shrinkage of the Transferred Epitaxial BaTiO_3_ Thin Films

**Figure 3:**
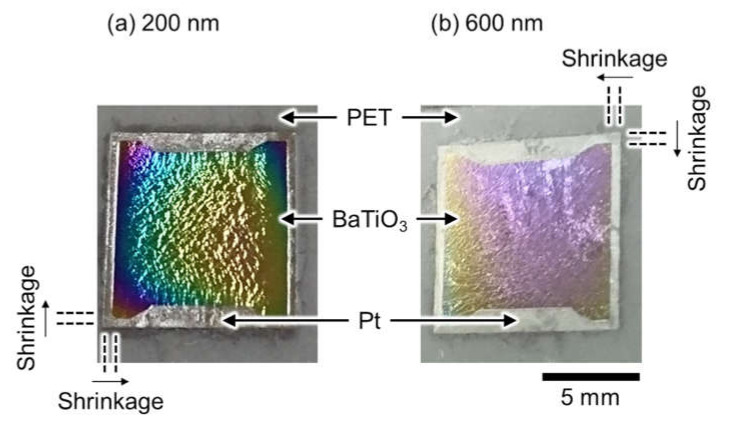
Photographs of the epitaxial BaTiO_3_ thin films after transfer of (**a**) 200- and (**b**) 600-nm-thick BaTiO_3_ thin films to PET sheets. The pairs of parallel dashed lines near the corners of the BaTiO_3_ thin films serve as visual guides for the drastic shrinkage of the epitaxial BaTiO_3_ thin films.

**Figure 4:**
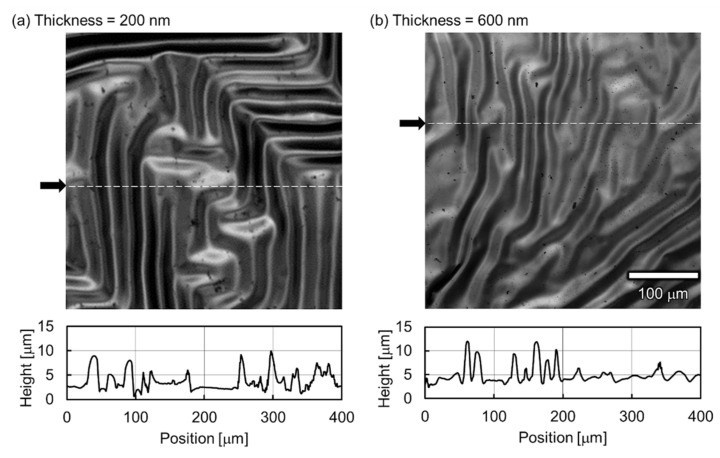
CLSM images (**top**) and section profiles (**bottom**) of (**a**) 200- and (**b**) 600-nm-thick epitaxial BaTiO_3_ thin films after transfer to PET sheets. The positions of the section profiles are indicated by arrows on the left-hand sides of the images. From both the images and profiles, the giant wrinkles with micrometer-order height can be clearly observed.

The transferred epitaxial BaTiO_3_ thin films showed a curious feature:their sizes. The sizes of the epitaxial BaTiO_3_ thin films were clearly smaller than the Pt buffer layers, after the transfer for thicknesses of 200 and 600 nm (Figure 3a,b, respectively). The parallel pairs of dashed lines in Figure 3 are drawn as visual guides. The original sizes of the epitaxial BaTiO_3_ thin films and the Pt buffer layers were both 10 mm × 10 mm, which were the same size as before the transfer from a 10 mm × 10 mm MgO(100) single-crystal surface (Figure 1). However, the sizes of the epitaxial BaTiO_3_ thin films measured with a vernier caliper were 8.9 mm × 8.9 mm after the transfer process (the sizes are independent of the thickness of the epitaxial BaTiO_3_ thin films), although the Pt buffer layers were still 10 mm × 10 mm after the transfer process. This indicates a drastic shrinkage of the epitaxial BaTiO_3_ thin films by ca. 10% for each side of the square thin film (the shadow of the sample holders during PLD of the epitaxial BaTiO_3_ thin films were observed as trapezoid shapes on the top and bottom domains in the images in Figure 3), even though BaTiO_3_ is a brittle ceramic. The possible origin of this remarkable shrinkage is observed in the CLSM images (top, Figure 4). The surface images (top, Figure 4) and the height profiles (bottom, Figure 4) of both the 200- and 600-nm-thick samples showed giant wrinkles. A feature of the giant wrinkles is their height, which were in the order of micrometers, which is surprisingly large compared to the thicknesses of the epitaxial BaTiO_3_ thin films (200 and 600 nm) and the Pt buffer layer (30 nm).

We consider that the giant wrinkles are formed not on the Pt buffer layers, but on the epitaxial BaTiO_3_ thin films, meaning that the epitaxial BaTiO_3_ thin films slipped on the unchanged Pt buffer layers during the drastic shrinkage and formation of the giant wrinkles. If the Pt buffer layers formed such giant wrinkles, the Pt buffer layers would shrink like the epitaxial BaTiO_3_ thin films, because the volume was not sufficient for the 30-nm-thick Pt buffer layer to form giant wrinkles with micrometer-order height without drastic shrinkage. The consideration suggests that there will be voids between the epitaxial BaTiO_3_ thin films and the Pt buffer layer. In future studies, a section of the sample will be investigated through a combination of scanning electron microscopy and energy dispersive X-ray spectroscopy to verify if there are any voids between the epitaxial BaTiO_3_ thin films and the Pt buffer layer.

The giant wrinkles on the epitaxial BaTiO_3_ thin films, accompanied by a drastic shrinkage of ca. 10%, means the application of an in plane compressive strain to the epitaxial BaTiO_3_ thin films. This compressive strain does not originate from the recovery of the thermal expansion after cooling down from the temperature of the heated etchant (40 °C) to room temperature, because the relative thermal expansion at room temperature is too small (Section 3.1). The results of our previous study [13] ruled out that the effect was caused by the Pt buffer layer, because an epitaxial SrTiO_3_ thin film transferred to a polyethylene naphthalate without a Pt buffer layer also showed small wrinkling. A possible origin of the giant wrinkles is the epitaxial tensile strain from MgO(100) (5.23%). Before transfer, the epitaxial BaTiO_3_ thin film was stretched in plane from the MgO(100) single-crystal substrate. The transfer process with the etching of the MgO(100) single-crystal substrate releases the epitaxial BaTiO_3_ thin film from this 5.23% tensile strain. The release of the tensile strain will cause giant wrinkling and result in the drastic shrinkage of the thin film. The Pt buffer layer will support the giant wrinkles accompanied by drastic shrinkage, because the prevention of the exfoliation of epitaxial BaTiO_3_ thin films accumulates tensile strain over a long distance. The detailed structure, such as the grain size of the epitaxial BaTiO_3_ thin films, will play an important role in this wrinkling process, while the detailed structure of the epitaxial BaTiO_3_ thin films is not currently understood. In the next step, we will study this subject.

The giant wrinkling is similar to the results of the pre-strain technique, where thin films with wavy structures deposited on pre-stretched elastomer have been reported [27,28,29]. The pre-strain technique is popular in the field of stretchable devices, so we will develop our finding of giant wrinkles in the field of *stretchable oxide electronics*. Stretchable devices have a large advantage for the use of wearable devices on the complicated shapes of the body surface to detect a variety of biosignals, i.e., *wearable biosensors*. It is important to understand the formation process of the giant wrinkles to realize this aim. Hence, real-time observation during the etching process of the MgO(100) single-crystal substrate will be performed, i.e., the formative process of the giant wrinkles in the next step. The evaluation of the ferroelectric and piezoelectric properties of the transferred epitaxial BaTiO_3_ thin films is also important to realize wearable biosensors.

## 4. Summary

We found that introducing a Pt buffer layer is effective for improving transfer of an epitaxial BaTiO_3_ thin film to a PET sheet, which resulted in the successful transfer of a 10 mm × 10 mm epitaxial BaTiO_3_ thin film without any serious damage. The ductile nature of Pt is expected to relax the large difference between the relative thermal expansion of BaTiO_3_ and PET and the thermal release tape (made of PMMA). This is important progress to realize flexible oxide engineering. The improvement of the transfer process of an oxide thin film with a larger area than previous reports showed two surprising results: drastic shrinkage of ca. 10% and giant wrinkles with micrometer-order height on the surface of the transferred epitaxial BaTiO_3_ thin films. The giant wrinkles seemed to be the principal source of the drastic shrinkage of the epitaxial BaTiO_3_ thin films. A possible source of the giant wrinkling could be the relaxation of the epitaxial tensile strain from the original MgO(100) single-crystal substrate during dissolution of MgO in a phosphoric acid aqueous solution. Such giant wrinkles accompanied by drastic shrinkage are similar to the results of the pre-strain technique in the fabrication process of stretchable devices. Therefore, it is expected that the formation of such giant wrinkles, accompanied by drastic shrinkage of the epitaxial BaTiO_3_ thin film, can be directly applied to the functional oxides of stretchable devices, such as wearable biosensors.

## Data Availability

The data that support the findings of this study are available from the corresponding author (H.N.) upon reasonable request.
